# Structural Analysis Reveals Conformational Plasticity in the Recognition of RNA 3′ Ends by the Human La Protein

**DOI:** 10.1016/j.str.2008.02.021

**Published:** 2008-06-11

**Authors:** Olga Kotik-Kogan, Elizabeth R. Valentine, Domenico Sanfelice, Maria R. Conte, Stephen Curry

**Affiliations:** 1 Biophysics Section, Blackett Laboratory, Imperial College, Exhibition Road, London SW7 2AZ, United Kingdom; 2 Randall Division of Cell and Molecular Biophysics, King's College London, New Hunt's House, Guy's Campus, London SE1 1UL, United Kingdom

**Keywords:** PROTEINS, RNA

## Abstract

The eukaryotic La protein recognizes the 3′ poly(U) sequences of nascent RNA polymerase III transcripts to assist folding and maturation. The 3′ ends of such RNAs are bound by the N-terminal domain of La (LaNTD). We have solved the crystal structures of four LaNTD:RNA complexes, each containing a different single-stranded RNA oligomer, and compared them to the structure of a previously published LaNTD:RNA complex containing partially duplex RNA. The presence of purely single-stranded RNA in the binding pocket at the interface between the La motif and RRM domains allows significantly closer contact with the 3′ end of the RNA. Comparison of the different LaNTD:RNA complexes identifies a conserved set of interactions with the last two nucleotides at the 3′ end of the RNA ligand that are key to binding. Strikingly, we also observe two alternative conformations of bound ssRNA, indicative of an unexpected degree of plasticity in the modes of RNA binding.

## Introduction

The La protein is a highly abundant nuclear phosphoprotein that is conserved in eukaryotes. Originally identified as an autoantigen associated with rheumatic diseases such as lupus erythematosus and Sjögren's syndrome, La has been shown to have key roles in the processing and use of coding and noncoding RNAs (Maraia and Intine, 2001; Wolin and Cedervall, 2002). Perhaps the best-characterized activity of the protein is the specific recognition of the oligo(U) sequences that are a distinguishing feature of the 3′ ends of most pol III precursors (e.g., pre-tRNA, 5S rRNA, and U6 and RNase P snRNAs) (Stefano, 1984; Maraia and Intine, 2001, 2002; Wolin and Cedervall, 2002). Binding of La prevents degradation by 3′ exonucleases (Yoo and Wolin, 1997; Fan et al., 1998) but can also contribute to the nuclear retention of certain RNA precursors (Intine et al., 2002; Bayfield et al., 2007), and is required for the normal pathway of pre-tRNA maturation (Van Horn et al., 1997; Fan et al., 1998; Chakshusmathi et al., 2003) and the proper assembly of other pol III transcripts, such as U6 RNA, into functional RNA:protein complexes (Xue et al., 2000).

In yeast, La has also been shown to bind to the 3′ poly(U) ends that become exposed during endonucleolytic processing of pol II transcripts (e.g., snRNAs), again serving to stabilize the RNAs against exonucleolytic nibbling (Kufel et al., 2000; Xue et al., 2000).

In addition to its roles in the maturation of noncoding transcripts, La can directly facilitate translation of specific cellular (Holcik and Korneluk, 2000; Intine et al., 2003; Trotta et al., 2003; Inada and Guthrie, 2004) and viral mRNAs (Craig et al., 1997; Ali et al., 2000; Costa-Mattioli et al., 2004), usually by binding *internal* RNA sequences within the untranslated regions of target mRNAs. Thus, La exhibits remarkable versatility in its modes of binding to RNA.

Human La (hLa) is a 408 amino acid monomeric modular protein containing three structured domains: a La motif and two RNA recognition motifs (RRMs 1 and 2); these are followed by a largely unstructured C-terminal tail that possesses a short basic motif (SBM) and a nuclear localization signal (NLS) (Maraia and Intine, 2001; Wolin and Cedervall, 2002). In recent years, investigations of the molecular basis of La:RNA interactions have focused on the N-terminal domain (NTD), the most strongly conserved region of the protein between all eukaryotic species. The LaNTD contains the La motif and RRM1, the domains required for high-affinity binding of 3′ oligo(U) RNA (Kenan, 1995; Goodier et al., 1997; Jacks et al., 2003; Alfano et al., 2004); indeed, it has been shown that the two domains must act synergistically to bind 3′ oligo(U) tails (Goodier et al., 1997; Ohndorf et al., 2001; Alfano et al., 2004; Dong et al., 2004).

Structural studies on La have so far revealed several surprising aspects of the molecule. Although initially predicted to adopt an RRM fold (Kenan, 1995), the controversial nature of the La motif domain (Maraia and Intine, 2001; Wolin and Cedervall, 2002) was finally settled when it was shown to be an elaborated version of the winged-helix domain (Alfano et al., 2004; Dong et al., 2004). Strikingly, chemical shift mapping experiments revealed that the recognition helix and wing loop of the La motif, which are normally used by this type of module to bind DNA (Gajiwala and Burley, 2000) or RNA (Yoshizawa et al., 2005), are not involved in recognition of 3′ oligo(U) sequences, indicating that La adopts a novel mode of interaction with RNA ligands (Alfano et al., 2004). Although the same approach suggested that the β sheet surface of RRM1 was also involved in 3′ end recognition, this has been challenged by a crystallographic study of the complex of the LaNTD with a nonameric 5′-UGCUGUUUU-3′ RNA oligomer (Teplova et al., 2006). The crystal structure revealed a number of important structural details of the recognition of the 3′ end of the RNA oligomer that help to explain the specificity of the LaNTD for sequences terminating in UUU_OH_. Although largely consistent with previous structural and mutagenic analyses (Alfano et al., 2004; Dong et al., 2004), in particular showing that a conserved Asp residue (D33) located in the interdomain cleft makes specific interactions with the 2′ and 3′ OH groups of the 3′ nucleotide, the bound RNA was observed to make only one H bond to a backbone amide at the very edge of the RRM1 β sheet. Surprisingly, and in contrast to all previous observations of RRM:RNA interactions (Maris et al., 2005), there were no contacts between the RNA and the β sheet surface of RRM1.

However, an unexpected consequence of the molecular packing observed in the LaNTD:RNA crystals complicated the interpretation of the structure (Curry and Conte, 2006; Maraia and Bayfield, 2006; Teplova et al., 2006). Due to crystal contacts, the RNA oligomer used in the experiment formed a 5 base pair duplex with a one-nucleotide overhang at the 5′ end and a three-nucleotide overhang at the 3′ end. Although physiological ligands for La such as pre-tRNA and Y RNA possess similar features, in particular having a 3′ oligo(U) overhang extending from base-paired RNA (van Gelder et al., 1994; Lee et al., 1997; Farris et al., 1999), they also exhibit notable differences from the RNA ligand in the crystal structure (Teplova et al., 2006). For example, the synthetic RNA used in the cocrystal had a 5′ hydroxyl group, but it is important to note that physiological ligands possess a 5′ triphosphate; because the 5′ end of the RNA in the cocrystal structure is in contact with the protein, it is difficult to predict the impact of the presence of a 5′ triphosphate on the conformation of bound RNA. In any case, it is still not clear what role the LaNTD may play in recognition of 5′ ends of RNA ligands. In human La, the SBM within the C-terminal region is required for the recognition of 5′ triphosphates (Fan et al., 1998; Bhattacharya et al., 2002); however, the relative disposition of the La motif and the SBM during this interaction is still unknown.

Thus, although the published structure represents a significant advance in our understanding of La:RNA interactions, the picture remains incomplete. To further advance the investigation of the structural basis of 3′ end recognition by La, we have cocrystallized LaNTD with four different short RNA oligomers. In each case, we used RNA oligonucleotides that were designed to remain single stranded in the crystals, allowing us to focus on interactions that are not constrained by base-pairing interactions. We find that the absence of duplex RNA in the binding pocket located at the interface between the La motif and RRM1 domains allows a significantly more intimate contact between the protein and RNA, which results from the two domains clamping more tightly onto the RNA 3′ end. Comparison of the variety of LaNTD:RNA complexes obtained reveals a conserved set of interactions that are focused on the last two nucleotides at the 3′ end of the RNA ligand. Strikingly, however, we also observe at least two alternative conformations of the 3′ ends of bound ssRNA, neither of which is precisely identical to the bound conformations reported previously (Teplova et al., 2006), suggesting that there is an unusual degree of plasticity in the allowable modes of RNA binding.

## Results

### Overall Structure of LaNTD Complexed with Different RNA Oligomers

**Figure 1:**
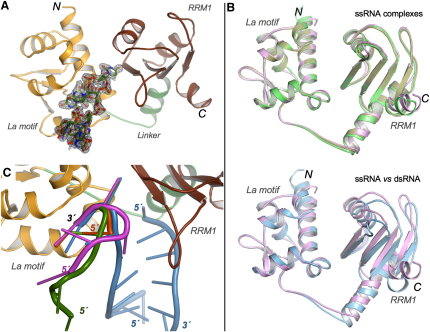
Structure and Comparison of LaNTD:RNA Complexes (A) Simulated annealing F_o_ − F_c_ omit map contoured at 1.75σ showing density for the AUAAUUU RNA oligomer bound to LaNTD. The protein is colored by domain: La motif, orange; interdomain linker, green; RRM1, brown. All structural figures were prepared using PyMOL (DeLano, 2002). (B) Comparison of LaNTD complexes with ssRNA and dsRNA, superposed using residues of the La motif. Top: the superposition of LaNTD complexes with the ssRNAs AUUUU (pink), AUAAUUU (green), and UUUUUUUU (brown) reveals a high degree of similarity. Bottom: superposition of LaNTD complexed with AUUUU (pink) and the self-annealing duplex RNA UGCUGUUUU (blue) reveals that the dsRNA ligand displaces RRM1 away from the La motif, increasing the width of the interdomain cleft. (C) Superposition of LaNTD:RNA complexes showing variation in the conformations of bound RNA. The protein is colored as in (A). The RNA oligomers shown are AUUUU (magenta), AUAAUUU (green), UUUUUUUU (orange), and the duplex of UGCUGUUUU (blue). Note that only the last two U's of UUUUUUUU are included in the model.

RNA oligomers for cocrystallization with LaNTD were selected to resemble the 3′ end sequence of pre-tRNA^Met^ (Harada et al., 1984) (AUAAUUU, AUAUUUU, AUUUU) or oligouridylate RNAs (UUUUUUUU) that were used in earlier NMR and binding experiments (Alfano et al., 2004). Diffraction-quality crystals for the four LaNTD:RNA complexes were obtained—in three different space groups—allowing the structures to be determined and refined to resolutions of between 1.8 and 2.8 Å (Experimental Procedures; Table 1; Figure 1).

In each of the four structures, the RNA ligand binds in a single-stranded conformation within the cleft between the La motif and RRM1 domains. The overall structures of the individual domains are essentially unchanged from their conformations in the absence of an RNA ligand (Alfano et al., 2004). However, amino acids 102–110 of the linker polypeptide connecting the domains (residues 93–110) invariably fold into an α helix, irrespective of the packing environment of the crystal (Figure 1B). Crucially, NMR experiments also show that residues 102–110 adopt this α-helical fold in solution but only when RNA binds: analyses of the chemical shift of backbone atoms and NOE patterns clearly indicate that, whereas the linker is largely unstructured in the apo-protein (apart from a single turn of 3_10_ helix in residues 107–109), it adopts an α-helical fold (residues 102–110) in the presence of short RNA oligomers (e.g., UUUU, UUUUUUUUUU) (Figure S2, see the Supplemental Data available with this article online). Together, these observations strongly suggest that formation of this helix in the interdomain linker is a conserved feature of La:RNA complexes.

Superposition of the four complexes using just the residues from the La motif domain shows that the RRM1 domains also superpose closely, thus indicating that there is a conserved relative disposition of the two domains when bound to ssRNA (Figure 1B). Strikingly, superposition of the LaNTD:ssRNA complexes with the La:RNA complex formed using partially double-stranded RNA (Teplova et al., 2006) reveals that the presence of dsRNA increases the separation of the La motif and RRM1 domains by 3–4 Å (Figure 1B). This occurs because of the added bulk of the RNA duplex; in particular, the 5′ end of the RNA strand that is base paired to the RNA oligomer which is bound to the LaNTD by its 3′ end sterically hinders the close approach of RRM1 to the La motif (Teplova et al., 2006) (Figure 1C). Moreover, this complementary strand perturbs the structure of the interdomain interface by displacing the side chains of F28 and R32 from the conformations that they are free to adopt in the presence of ssRNA (Figure S1). The absence of this complementary strand in the LaNTD:ssRNA complexes reported here allows a fully intimate set of contacts to form between both domains and the 3′ end of the RNA (see below for details).

### Comparison of RNA Binding Modes

Superposition of all five crystal structures of LaNTD:RNA complexes reveals the conserved structural features of the protein:RNA interactions, particularly at the 3′ ends, but also shows that there is considerable variation in the conformations of the 5′ ends of the RNA ligands (Figure 1C). Because the RNAs used in these structures have different lengths but show most commonalities at their 3′ ends, we have adopted a negative numbering system for the nucleotide residues, beginning at −1 for the 3′ nucleotide, −2 for the preceding nucleotide, and so on, toward the 5′ end.

**Figure 2:**
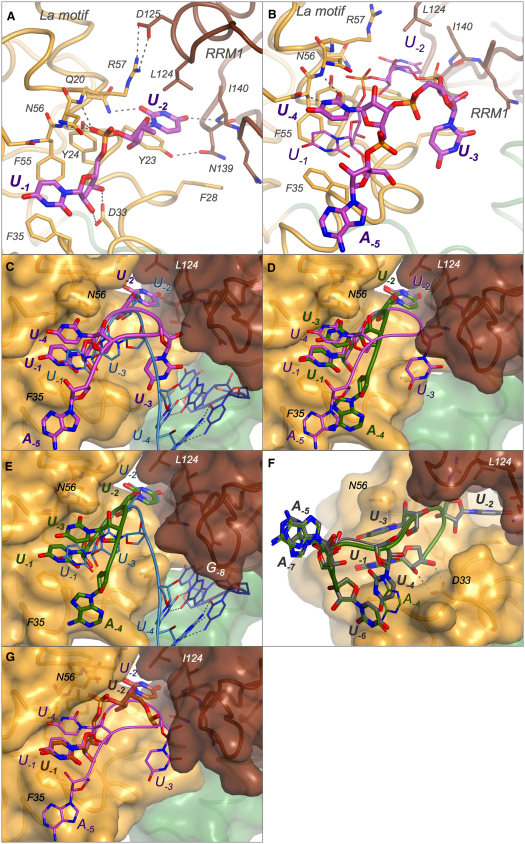
Comparative Details of ssRNA and dsRNA Bound to LaNTD (A) Structure of the LaNTD:AUUUU complex showing just the last two nucleotides, U_−1_ and U_−2_. The RNA is shown as a stick model with carbon atoms colored magenta. The protein is colored by domain as in Figure 1A; selected side chains are shown as sticks with their carbon atoms colored by domain. Other atoms are colored by type: oxygen, red; nitrogen, blue; phosphorus, orange. Hydrogen bonds are shown as dashed lines. (B) Structure of the LaNTD:AUUUU complex shown with all five nucleotides; U_−1_ and U_−2_ are depicted with thinner lines. (C–G) Superpositions of different RNA ligands; in each case, the protein structure shown (surface colored by domain) is that for the RNA depicted in thick lines. The RNA backbone, shown as a tube, and carbon atoms are colored as in Figure 1C. (C) AUUUU (magenta) compared to the dsRNA UGCUGUUUU (blue). (D) AUUUU (magenta) compared to AUAAUUU (green). (E) AUAAUUU (green) compared to the dsRNA UGCUGUUUU (blue). (F) AUAAUUU (green) compared to AUAUUUU (gray). (G) UUUUUUUU (orange) compared to AUUUU (magenta).

The binding conformation of the terminal two bases, U_−1_ and U_−2_, is essentially identical in all the structures. U_−1_ interacts exclusively with the La motif (Figure 2). Intriguingly, specific H-bond interactions between U_−1_ and the La motif only involve *backbone* features of the nucleotide: the 2′ and 3′ OH groups from the ribose ring of U_−1_ are specifically recognized by hydrogen bonds to the O atoms of the side-chain carboxylate moiety of D33 (Figure 2A), interactions that were observed previously (Teplova et al., 2006); additionally, the O1 atom of the U_−1_ phosphate group makes hydrogen bonds with both the backbone NH groups of N56 and R57 and with the side-chain OH of Y24. Consistent with the structure, these interactions with the ribose-phosphate backbone have been shown by mutagenesis to be important for 3′ end recognition (Dong et al., 2004; Teplova et al., 2006).

The U_−1_ base of ssRNA ligands stacks directly onto the side chain of F35, as reported previously (Teplova et al., 2006). Although it packs closer into the body of the La motif than observed for the partial dsRNA ligand (by at least 1 Å), making apolar contacts with K54 and F55, it makes no base-specific interactions with the protein (Figures 2A and 2C). The structures show that because only one side of the base contacts the La motif, while the other faces the solvent, there is room to accommodate other types of base, even larger purines; this is consistent with binding measurements showing that variation of the terminal base has only a small impact on binding affinity (Teplova et al., 2006).

The penultimate nucleotide, U_−2_, makes intimate contacts with the protein that involve both the La motif and RRM1 (Figure 2A). In contrast to U_−1_, it is the nucleotide base of U_−2_ and not its backbone that makes specific hydrogen bonds to the protein. Atoms O2 and O4 from the pyrimidine ring of U_−2_ hydrogen bond to the side-chain amide of Q20 (La motif) and the main-chain amide of I140 (RRM1), respectively, a pair of interactions that clearly helps to draw the two domains together. Notably, both these hydrogen bonds are significantly shorter in the presence of ssRNA ligands (∼2.8 Å) compared to the interactions observed when partial dsRNA is bound (∼3.1 Å) (Teplova et al., 2006), illustrating again that ssRNA ligands permit a closer association with the protein. The pyrimidine ring of U_−2_ is also stacked directly on top of Y23, a residue from the La motif that is positioned by a hydrogen bond from the tyrosine side chain to the side chain of N139 of RRM1, which in turn stacks on top of F28 from the La motif (Figure 2A). This latter stacking interaction was not observed in the presence of dsRNA because the side chain of F28 was forced out of position by steric hindrance owing to the complementary strand of the duplex RNA (Teplova et al., 2006). The U_−2_ pyrimidine ring is also packed directly underneath L124 from RRM1, a residue that is at least partly secured in place by the salt bridge made by its neighbor (D125) with R57 from the La motif. Thus, the recognition of U_−2_ in the context of ssRNA engages a concerted set of protein:RNA and protein:protein interactions involving residues from *both* domains to form a tightly defined pocket that is specific in size, shape, and hydrogen-bonding capacity for a uridylate base (Figure 2C). NMR analyses show that these interdomain interactions only form upon RNA binding (see below). This induced fit of the binding pocket around U_−2_ accounts well for the cooperative nature of RNA binding by both domains of LaNTD (Goodier et al., 1997; Ohndorf et al., 2001; Alfano et al., 2004; Dong et al., 2004).

In the previously published structure, the 3′ end of the RNA was found to make a tight turn to allow the U_−3_ base to stack onto U_−1_ in an offset or half-staggered conformation. In the complexes formed with ssRNA, U_−1_ is also found to be stacked underneath an RNA base, but there are notable differences in the way that this is achieved by different RNA oligomers.

For the sequence AUUUU, the bound RNA oligomer follows a wider turn such that U_−4_ rather than U_−3_ stacks on U_−1_. In this case, U_−3_ is flipped out and makes no specific contacts with the protein (Figures 2B and 2C). The U_−4_ base stacks directly above U_−1_, rather than in the offset manner observed previously (Teplova et al., 2006). Moreover, because the U_−1_/U_−4_-stacked pair pack closer to the La motif (probably because the ssRNA ligand is under fewer conformational constraints than the partial duplex RNA), this now allows U_−4_ to make a base-specific hydrogen bond with the backbone of the La motif (Figure 2B). Thus, atoms O2 and N3 from the U_−4_ pyrimidine ring are bonded to the carbonyl oxygen of N54 and the amide hydrogen of N56, respectively; moreover, the side chain of N56 makes van der Waals contacts with the U_−4_ base. None of these interactions were observed with the partial dsRNA ligand (Teplova et al., 2006).

Intriguingly, when LaNTD was complexed with an ssRNA oligo having just three uridylates at the 3′ end (AUAAUUU), a tight turn in the RNA backbone was observed (Figure 2D), allowing U_−3_ to stack on U_−1_ in a conformation similar to that in the structure obtained with partial dsRNA (Teplova et al., 2006). However, the U_−1_/U_−3_ stacking is significantly less offset with the ssRNA ligand (Figure 2D). In fact, it closely resembles the U_−1_/U_−4_ stacking observed for AUUUU and thus permits U_−3_ to make the same base-specific hydrogen bonds that were described above for U_−4_ in the AUUUU RNA oligomer. In the case of the partial dsRNA ligand, U_−3_ is pulled out of a full stacking interaction with U_−1_ because of the involvement of U_−4_ in base-pairing interaction with the complementary strand of the duplex (Figure 2E).

The LaNTD:RNA complexes involving AUUUU and AUAAUUU occur in different space groups, C222 and C2, respectively (Table 1), making it difficult to distinguish the effects of the RNA sequence and the local crystallographic packing environment on the conformation of the bound RNA (Figure S3). To try to address this issue, we cocrystallized LaNTD with AUAUUUU to obtain a complex with an RNA having four uridylates at the 3′ end in the same C2 space group as the complex with AUAAUUU RNA which only has three 3′ uridylates. We found that the two heptameric RNA oligomers bind with essentially identical conformations (Figure 2F). Thus, although AUAUUUU has four 3′ uridylates, it still adopts the tight turn observed for AUAAUUU so that U_−3_ is stacked on U_−1_. This finding suggests that the packing environment can have a considerable influence on the bound conformation. It also indicates that the interaction made by the stacked base (U_−3_ or U_−4_) may be relatively weak, a finding that is consistent with binding measurements (see below).

Cocrystallization of LaNTD with the octameric UUUUUUUU oligomer in a third space group (P2_1_2_1_2) further illustrated the potential impact of packing environment on the analysis of such complexes. In this case, although U_−1_ and U_−2_ were found to adopt the same conformations as observed in all other complexes (Figure 2G), the remainder of the RNA was largely disordered (and therefore not included in the refined model). Close inspection of the packing contacts revealed that, although the electron density suggests that U_−3_ may stack on U_−1_, it cannot do so at full occupancy owing to a steric clash with a symmetry-related RNA ligand within the crystal (Figure S3).

### NMR Analyses of LaNTD:RNA Interactions in Solution

Although a full solution structure determination of LaNTD:RNA complexes was hindered by the limited stability and solubility of the samples under our experimental conditions, NMR analyses of LaNTD:RNA complexes in solution nevertheless provided important additional insights into the molecular basis of RNA recognition that are consistent with the crystallographic observations discussed above.

**Figure 3:**
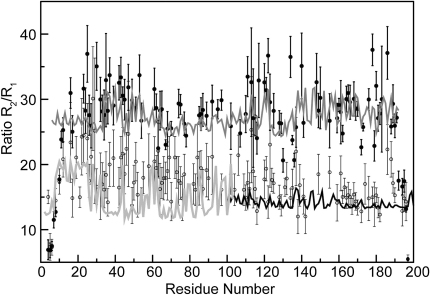
NMR Relaxation Measurements Reveal Rigidification of LaNTD upon RNA Binding The R_2_/R_1_ ratios for apo-LaNTD (open circles) and the LaNTD:UUUU complex (closed circles) are plotted against amino acid number. Also shown are HYDRONMR-predicted ratios for RNA-bound LaNTD (medium gray line) and the individual domains, the La motif (light gray line) and RRM1 (black line). Relaxation rates were calculated from peak intensity decay curves fit using CurveFit (http://cpmcnet.columbia.edu/dept/gsas/biochem/labs/palmer/). Uncertainties were calculated using jackknife simulations (Mosteller and Tukey, 1977).

In particular, transverse and longitudinal relaxation rate ratios (R_2_/R_1_) were used to obtain information about the dynamics of the La motif and RRM1 domains of LaNTD in the absence and presence of either UUU or UUUU RNA oligomers (Kay et al., 1989; Fushman et al., 1999). The measured average value of R_2_/R_1_ was around 27 for RNA-bound LaNTD (Figure 3), congruent with the R_2_/R_1_ ratio predictions performed with HYDRONMR (Bernado et al., 2002) using the coordinates of the LaNTD:AUAAUUU complex. In contrast, the observed average ratio for apo-LaNTD (around 17) is similar to that predicted for the individual domains (Figure 3). These data are consistent with a model in which the domains are able to tumble independently in solution in apo-LaNTD, whereas the binding of RNA stabilizes a fixed conformation of the component domains. This is also in agreement with the finding that the crystal structures of different LaNTD:ssRNA complexes have the same overall conformation (Figure 1B).

HSQC experiments were performed to observe chemical shift perturbations associated with RNA binding to LaNTD using a range of RNA oligomers (UU, UUU, UUUU, UCUU, AUUUU, UUUUUUUUUU) (Figure S4). Although a fully detailed analysis cannot be made due to spectral overlap, it is notable that very similar patterns of chemical shift perturbations were observed in all cases, consistent with a common mode of 3′ end recognition that involves both the La motif and RRM1 domains and appears largely dominated by the interactions made by the last two nucleotides in the sequence, U_−1_ and U_−2_, as found in the crystal structures. Intriguingly, comparison of UCUU with oligo(U) ligands shows some small differences in the chemical shifts of the residues lining the binding cleft (32–38, 55–60, and 140–145), suggestive of an alternative binding conformation (involving U_−4_/U_−1_ rather than C_−3_/U_−1_ stacking). The complex with UU also revealed a few minor differences in chemical shifts of the protein amide resonances in the binding pocket compared to longer oligo(U) ligands; this is likely to be because of the absence of uridylate residues preceding U_−2_ in this oligomer. Moreover, the spectrum obtained with AUUUU exhibits splitting of several resonances associated with residues in and around the binding site (32–34, 38, 55–60, and 140–145), suggestive of a slow conformational equilibrium between two states, possibly reflecting the two modes of binding observed in the crystal structures (Figure S2).

### RNA Binding Affinities

**Figure 4:**
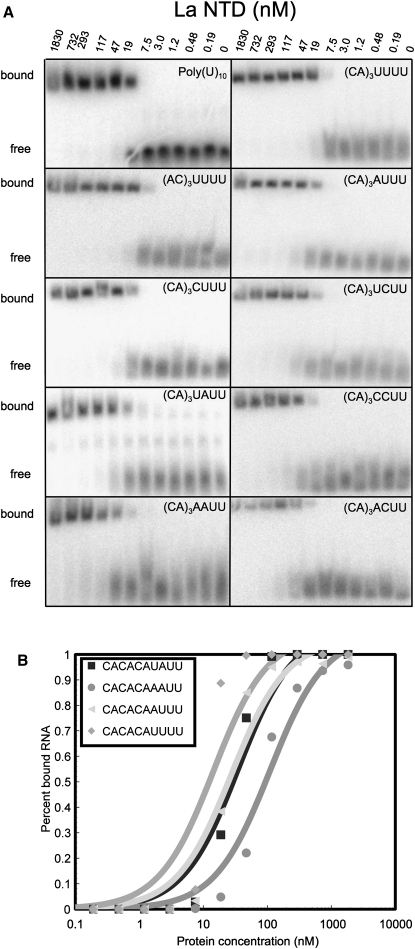
Gel-Shift Analysis of LaNTD:RNA Interactions (A) Autoradiograms of the gel analysis of radiolabeled RNA oligomers. Details are given in Experimental Procedures. Protein concentration is shown on the top and the RNA oligomer used in each experiment is indicated within each panel. (B) Sample fits of the binding data are shown for CACACAUUUU, CACACAAUUU, CACACAUAUU, and CACACAAAUU. Binding curves are shown for selected RNA oligomers.

In spite of the variations associated with different packing environments, it is important to emphasize that for RNA sequences terminating in four uridylates, two distinct modes of RNA binding to LaNTD are possible, involving either U_−1_/U_−3_ or U_−1_/U_−4_ stacking interactions. The variation observed suggests that, although U_−3_ or U_−4_ may make base-specific contacts with the La motif (see above), these may not contribute much to binding affinity. We probed this structural interpretation by measuring the binding affinities of RNA decamers containing different sequences of the four nucleotides at their 3′ ends. Strikingly, although the binding affinity did indeed increase with the number of uridylates at the 3′ end (4 > 3 > 2), the benefit of adding a third or fourth uridylate was rather modest (Figure 4; Table 2). Thus, the binding energy appears to be largely derived from the interactions made by the terminal two uridylates, a finding that accords well with previous binding data (Stefano, 1984; Teplova et al., 2006) and with the observation that these nucleotides make the most structurally conserved interactions with the protein (Figure 2). Addition of a single uridylate at the −3 or −4 position enhances the affinity by about 2-fold, presumably because of the ability of this nucleotide to base stack on U_−1_ and make hydrogen-bond interactions with the backbone of the La motif. There is a slight further enhancement of affinity if *both* the −3 and −4 positions are occupied by uridylates, even though the structure shows that only three of the four bases make contact with the protein. The affinity increase observed with four 3′-terminal uridylates appears to derive from the fact that the RNA in the complex can adopt either of *two* conformations that allow U_−3_ or U_−4_ to stack on U_−1_ and hydrogen bond to the La motif; the entropic penalty of binding is thereby likely to be reduced.

## Discussion

The comparative crystallographic analysis of different protein:RNA complexes provides a much fuller picture of the allowable modes of RNA binding of LaNTD. In cases such as this, where a significant portion of the RNA ligand extends from the body of the protein and is unavoidably involved in crystal-packing interactions, it is useful to be able to examine a variety of different complexes, because this helps to mitigate the possible effects of crystal packing on the bound conformation of the RNA. Comparison of the structures of the four LaNTD:RNA complexes reported here with the previously published crystal structure (Teplova et al., 2006) confirms and expands our understanding of the conserved features of 3′ end recognition by La.

The major interactions are made by the terminal pair of nucleotides (U_−1_/U_−2_), which consistently make the closest contacts with the protein and appear to provide the majority of the binding energy for the interaction. Of these two nucleotides, only U_−2_ makes base-specific contacts with the protein; U_−1_ certainly contributes to binding but does so in a base-independent manner, by stacking on F35 and hydrogen bonding via backbone hydroxyl and phosphate groups to the La motif (Figure 2A). Beyond the terminal pair of uridylates, we observe significant variation in the disposition of the rest of the 3′ end of the RNA, which allows either U_−3_ or U_−4_ to add modestly to the binding affinity. Indeed, the LaNTD evidently allows RNA ligands to exhibit considerable plasticity in the bound state; thus, RNAs containing base-paired segments with a 3′ overhang can bind, even though the duplex may restrict the LaNTD from clamping down fully on the 3′ end of the RNA (Teplova et al., 2006) (Figures 1B and 2C).

The structural results account well for the binding data reported here and in earlier studies. Although three or four uridylates at the 3′ end of the RNA have consistently been shown to confer optimal binding affinity, a strong interaction can be made with just two terminal uridylates (Stefano, 1984; Huang et al., 2005; Teplova et al., 2006) (Figure 4; Table 2). Indeed, the identity of the terminal base is not crucial for binding, because substitution by A or G reduces the affinity only very slightly (Stefano, 1984; Teplova et al., 2006); substitution by C has the largest effect but still only reduces the affinity by 3-fold (Teplova et al., 2006). U_−2_ evidently makes the most important *base-specific* contribution to binding affinity—base substitutions reduce binding by at least 10-fold (Teplova et al., 2006); this is entirely consistent with the structural observations that U_−2_ makes the most intimate contacts with the protein, indeed inducing the La motif and RRM1 domains to come together to form a snug pocket around the nucleotide side chain (Figure 2).

Curiously, it has been found that *Schizosaccharomyces pombe* La (sLa) requires at least four uridylates at the 3′ end for high-affinity binding (Huang et al., 2005). Although it is not entirely clear from the structure why this is so, in part because of the presence of an ∼60 amino acid N-terminal extension in sLa that does not occur in hLa, examination of the binding interface of the LaNTD:RNA complexes shows that interactions contributing to the cooperative binding of RNA 3′ ends by the La motif and RRM1 may be impaired in sLa owing to amino acid substitutions. Thus, the substitutions Y23F, N139A, and D125I in sLa (hLa residue numbering) eliminate the Y23-N139 and R57-D125 hydrogen-bond interactions between the La motif and RRM1 that are observed to be induced by RNA binding in hLa (Figures 2A and 2B). Moreover, the hydrophobic stacking of L124 on U_−2_ in hLa is disrupted by the L124Q substitution in sLa.

In none of the five LaNTD:RNA complexes now solved are the canonical RNA binding surfaces of component domains involved in interaction with RNA. Thus, neither the winged helix of the La motif nor the β sheet surface of RRM1 makes contact with the RNA. It remains possible that this observation is due to artifactual crystal-packing constraints, but this seems unlikely given the variety of RNA sequences used and the fact the complexes crystallized in four different space groups. The previous observation that a decameric oligouridylate RNA caused chemical shifts on the β sheet surface, indicative of possible direct contacts (Alfano et al., 2004), may be a result of the RNA-induced folding of the interdomain linker polypeptide which leads to formation of an α helix proximal to the β sheet surface of RRM1. This interpretation is supported by the observation that similar shifts were also recorded with a shorter UUUUU oligomer which would be unable to span the distance from the 3′ end binding site and the surface of RRM1 (Alfano et al., 2004). Curiously, the folding of the interdomain linker into a helix appears to be an indirect effect of RNA binding because there are no contacts between the RNA and this portion of the polypeptide (Figure 1).

**Figure 5:**
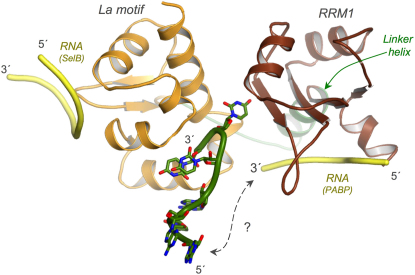
Model Showing Potential Additional RNA Binding Surfaces on LaNTD The crystal structure of the LaNTD:AUAAUUU complex is shown, with the protein colored by domain and the RNA depicted with green backbone and carbon atoms. The “canonical” modes of RNA binding to the La motif and RRM1 are indicated by yellow tubes which represent the backbone conformations of RNA ligands for SelB and the polyA binding protein (PABP). These ligands were positioned by superposing the SelB (Yoshizawa et al., 2005) and PABP (Deo et al., 1999) protein:RNA structures on the La motif and RRM1 domains of LaNTD, respectively. In each case, only part of the RNA ligand is shown. A possible connection between two segments from a large hypothetical RNA ligand is indicated by a dashed line.

RNA-induced formation of this helix may nevertheless affect the binding of larger ligands owing to its proximity to the putative RNA binding site on the β sheet surface of RRM1 and because more complex RNAs may be able to make simultaneous contacts with both the RRM1 β sheet and the interdomain cleft that binds the RNA 3′ end. Comparative modeling using known RRM:RNA structures (e.g., U2B″, PABP) shows that the RNA generally lies across the β sheet surface in a 5′-3′ direction that would place the 3′ end of the RNA close to the site in the interdomain cleft that recognizes this feature (Figure 5). Although such an interaction has not been seen with the short RNA oligomers used to date, longer RNAs may be able to bind simultaneously to the two binding sites; indeed, large RNA ligands might also be able to engage the winged helix of the La motif (Figure 5). Such a hypothesis is consistent with evidence that La can contact regions of RNA ligands beyond the 3′ end in pre-tRNA (Chakshusmathi et al., 2003; Copela et al., 2006) and that mutations in the β sheet of RRM1 can impair the ability of the protein to rescue misfolding pre-tRNA (Huang et al., 2006). However, it will require further structural investigation with larger La constructs that contain additional structural and functional features to address these questions.

## Experimental Procedures

### Plasmid Construction and Protein Purification

The N-terminal domain of human La protein (LaNTD, amino acids 4–194) was amplified from pET-La(1–408) using PCR and subcloned into a modified pETM-11 vector which adds a thrombin-cleavable N-terminal 6× His tag (Birtley and Curry, 2005). The protein was expressed at 37°C in *Escherichia coli* strain BL21 (DE3) for 3 hr after induction with 1 mM IPTG (isopropyl β-D-1-thiogalactopyranoside).

Cell pellets were resuspended in 25 mM Tris-HCl (pH 7.6), 250 mM NaCl. Protease inhibitor cocktail (Sigma) and 0.01% Triton X-100 were added immediately prior to sonication. The protein was purified from the soluble fraction of the cell lysates using TALON metal affinity resin (BD Biosciences). The N-terminal His tag was removed by incubating in the presence of 10 U Thrombin (Sigma) per mg of protein during dialysis against 100 mM KCl, 2 mM CaCl_2_, 50 mM Tris (pH 7.5). This leaves a Gly-Ser dipeptide at the N terminus of the processed protein. The cleaved His tag was removed by reapplying the sample onto TALON resin; the LaNTD protein was then loaded onto a 10 ml HiTrap heparin column (GE Healthcare) in 20 mM Tris-HCl (pH 7), 100 mM KCl, 1.5 mM MgCl_2_, 0.2 mM EDTA and eluted with a 0.1–2 M KCl gradient over ten column volumes. The purified protein was buffer exchanged into 20 mM Tris-HCl (pH 7), 50 mM NaCl, 1 mM MgCl_2_, 1 mM dithiothreitol and concentrated to 12.5 mg/ml.

The slightly longer form of LaNTD (La 1–194) used in NMR studies and gel binding assays was expressed and purified as described previously (Alfano et al., 2004).

### Crystallization and Data Collection

RNA oligomers (5′-AUUUU-3′, 5′-UUUUUUUU-3′, 5′-AUAAUUU-3′, 5′-AUAUUUU-3′) were synthesized, purified, and deprotected by Dharmacon Research. LaNTD:RNA complexes for crystallization were prepared at a molar ratio of 1:1. All crystallizations were performed using sitting-drop vapor diffusion. Crystals of the LaNTD:AUUUU complex grew spontaneously in 2.7 M ammonium sulfate, 0.1 M sodium citrate (pH 6.0) at 4°C; larger crystals (typically 0.4 × 0.2 × 0.1 mm^3^) grew within 2 weeks of streak seeding into similar drops with 2.6–2.8 M ammonium sulfate. LaNTD:UUUUUUUU complex crystals were grown in 0.1 M MMT buffer (0.02 M malic acid, 0.04 M MES, 0.04 M Tris [pH 4.6]), 32% (w/v) PEG 1500 at 20°C. Typical crystal dimensions were 0.3 × 0.4 × 0.2 mm^3^. LaNTD:AUAAUUU and LaNTD:AUAUUUU complexes were crystallized in 0.2 M NaCl, 0.1 M phosphate citrate (pH 5.0), 22% (v/w) PEG 8000, 0.01 M taurine. Because the two oligomers differ only by one nucleotide (A or U at the 5′ end), crystals of the LaNTD:AUAUUUU complex were obtained by streak seeding from LaNTD:AUAAUUU crystals. In both cases, crystals grew as thin needles with typical dimensions 0.7 × 0.05 × 0.02 mm^3^.

Immediately before flash cooling in liquid nitrogen, crystals were transferred briefly into a cryoprotectant solution consisting of mother liquor containing 30% (v/v) glycerol. X-ray diffraction data were collected at the European Synchrotron Radiation Facility, Grenoble, France.

### Structure Determination

Diffraction data were processed using the CCP4 package (CCP4, 1994). In every case, the data were phased by molecular replacement with Phaser (McCoy et al., 2007) using as a search model the La motif (residues 5–97) and RRM1 domain (residues 110–184) from the previously reported crystal structure (Teplova et al., 2006). The resulting electron density maps showed clear features for the RNA ligands; these were built into the atomic model and adjustments made to the protein structure using the program O (Jones et al., 1991). The structures were refined using CNS (Brunger et al., 1998) (Table 1).

### NMR Relaxation Measurements

Relaxation experiments (T_1_, T_2_, and {^1^H}-^15^N NOE) were acquired on a Bruker Avance at 14.1 T using the pulse sequences adapted from standard schemes (Kay et al., 1989). To determine conditions with minimal protein aggregation, transverse relaxation rates (R_2_) were measured both for apo- and RNA-bound protein at 0.6, 0.4, and 0.2 mM. Because the rates at the two lower protein concentrations were very similar (with and without RNA), all measurements were performed at 0.2 mM. R_2_ were measured with eight different total CPMG relaxation delays ranging from 6.36 to 82.68 ms. The longitudinal relaxation rates (R_1_) were measured with relaxation delays ranging from 10 to 1100 ms, typically in eight evenly spaced points. In both experiments, the shortest and the longest points were repeated for error calculations. Relaxation rates were calculated from peak intensity decay curves fit using CurveFit (http://cpmcnet.columbia.edu/dept/gsas/biochem/labs/palmer/). Uncertainties were calculated using jackknife simulations (Mosteller and Tukey, 1977). HYDRONMR (Garcia de la Torre et al., 2000; Bernado et al., 2002) predictions were performed using the radius length of 3.3 Å. The La motif and the RRM1 predictions were calculated using the coordinates from the first structure (Protein Data Bank [PDB] ID codes 1S7A and 1S70, respectively) (Alfano et al., 2004); the prediction for RNA-bound LaNTD was obtained using the protein coordinates from the LaNTD:AUAUUUU crystal structure.

### Gel-Shift Assays

RNA oligomers were synthesized and gel purified by Dharmacon. The assays were performed using protocols reported previously (Jacks et al., 2003). Each experiment was repeated between three and six times using freshly prepared protein.

Dissociation constants were determined by plotting the percent of bound RNA versus the protein concentrations. The curves were fit using the following equation (Irvine et al., 1991):

$$\phi =\frac{\left(P+N+K_{D}\right)-\sqrt{{\left(P+N+K_{D}\right)}^{2}-4PN}}{2N},$$

where *ϕ* is the fraction of bound RNA determined by the intensity of the protein-bound RNA signal divided by the total RNA signal (both bound and free), *P* is the protein concentration, and *N* is the RNA concentration. Theoretical fitting was performed using least-squares analysis. The RNA concentration was estimated to be 65% of the calculated concentration (taking into account the loss of material following spin column purification) and then fitted iteratively using least-squares analysis. Relative dissociation constants were calculated using CACACAAUUU as a reference point, assuming that this oligomer bound LaNTD in a single conformation. Typical RNA concentrations were around 1 nM.

## Figures and Tables

**Table 1:** Crystallographic Data Collection and Refinement Statistics

Data Collection				
RNA Oligomer	AUAAUUU	AUAUUUU	UUUUUUUU	AUUUU
Space group	C2	C2	P2_1_2_1_2	C222
Unit cell parameters	a = 141.62 Å	a = 140.03 Å	a = 98.31 Å	a = 96.07 Å
	b = 44.45 Å	b = 44.47 Å	b = 114.59 Å	b = 116.69 Å
	c = 91.58 Å	c = 92.29 Å	c = 37.41 Å	c = 63.51 Å
	α = 90°	α = 90°	α = 90°	α = 90°
	β = 114.22°	β = 114.35°	β = 90°	β = 90°
	γ = 90°	γ = 90°	γ = 90°	γ = 90°
X-ray source	ID23-1	ID23-1	ID14-1	ID23-1
Resolution range (Å)^a^	43−2.1 (2.21−2.10)	43−2.1 (2.21−2.10)	41−1.8 (1.9−1.8)	63−2.8 (2.95−2.8)
Unique reflections	27,637	28,656	39,488	8,894
Multiplicity^a^	1.7 (1.6)	2.7 (2.7)	3.4 (3.4)	2.4 (2.4)
I/σ_I_^a^	7.5 (4.1)	8.1 (3.0)	6.7 (1.8)	6.2 (1.9)
Completeness (%)^a^	90.4 (92.2)	95.2 (95.0)	98.7 (97.7)	98.3 (99.9)
R_merge_ (%)a,b	8.4 (22.3)	7.9 (41.3)	6.9 (40.5)	8.9 (37.8)

Refinement				

Residue range in LaNTD model	8–186	6–192	10–188	8–192
Number of protein and RNA atoms	3,311	3,340	3,028	1,540
Number of water molecules	208	164	201	0
R_work_/R_free_ (%)^c^	23.3/26.9	23.2/27.2	25.0/27.9	26.1/29.0
Root-mean-square deviation from ideal bond lengths (Å)	0.006	0.006	0.006	0.006
Root-mean-square deviation from ideal bond angles (°)	1.20	1.20	1.18	1.20
Ramachandran plot (% favored/allowed)	95.0/4.7	91.9/7.2	93.6/6.1	84.3/14.5
PDB ID code	2VON	2VOD	2VOO	2VOP

a Values for highest resolution shell are given in parentheses.

b R_merge_ = 100 × Σ_hkl_|I_j_(hkl) − < I_j_(hkl) >|/Σ_hkl_Σ_j_I(hkl), where I_j_(hkl) and < I_j_(hkl) > are the intensity of measurement j and the mean intensity for the reflection with indices hkl, respectively.

c R_work_ = 100 × Σ_hkl_||F_obs_| − k|F_calc_||/Σ_hkl_|F_obs_|, where k is a scale factor and R_free_ is the equivalent R factor for the test set of reflections.

**Table 2:** RNA Binding Data

RNA Oligomer	K_D_ (Relative)
UUUUUUUUUU	1.0 ± 0.4
CACACAUUUU	0.8 ± 0.3
ACACACUUUU	0.7 ± 0.2
CACACAAUUU	1.0
CACACACUUU	1.2 ± 0.4
CACACAUCUU	1.4 ± 0.5
CACACAUAUU	1.0 ± 0.2
CACACAACUU	2.0 ± 0.8
CACACACCUU	2.4 ± 0.5
CACACAAAUU	2.8 ± 1.1

K_D_ values shown are relative to that for CACACAAUUU (K_D_ = 30 nM).

## Accession Numbers

Coordinates for the complexes of LaNTD with AUAUUUU, AUAAUUU, UUUUUUUU, and AUUUU have been deposited in the Protein Data Bank under ID codes 2VOD, 2VON, 2VOO, and 2VOP, respectively.

## Supplemental Data

Document S1. Four Figures and Supplemental References
