# Supplementary material for: Structural Analysis Reveals Conformational Plasticity in the Recognition of RNA 3′ Ends by the Human La Protein
Source: Structure. 2008 Jun 11;16(6):852–62. doi: 10.1016/j.str.2008.02.021 (PMC2430598; doi:10.1016/j.str.2008.02.021)
Supplement: Document S1. Four Figures and Supplemental References [file mmc1.pdf]

**Structure *16***

# Supplemental Data

# Structural Analysis Reveals Conformational Plasticity in the Recognition of RNA 3′ Ends by the Human La Protein

**Olga Kotik-Kogan, Elizabeth R. Valentine, Domenico Sanfelice, Maria R. Conte, and Stephen Curry**

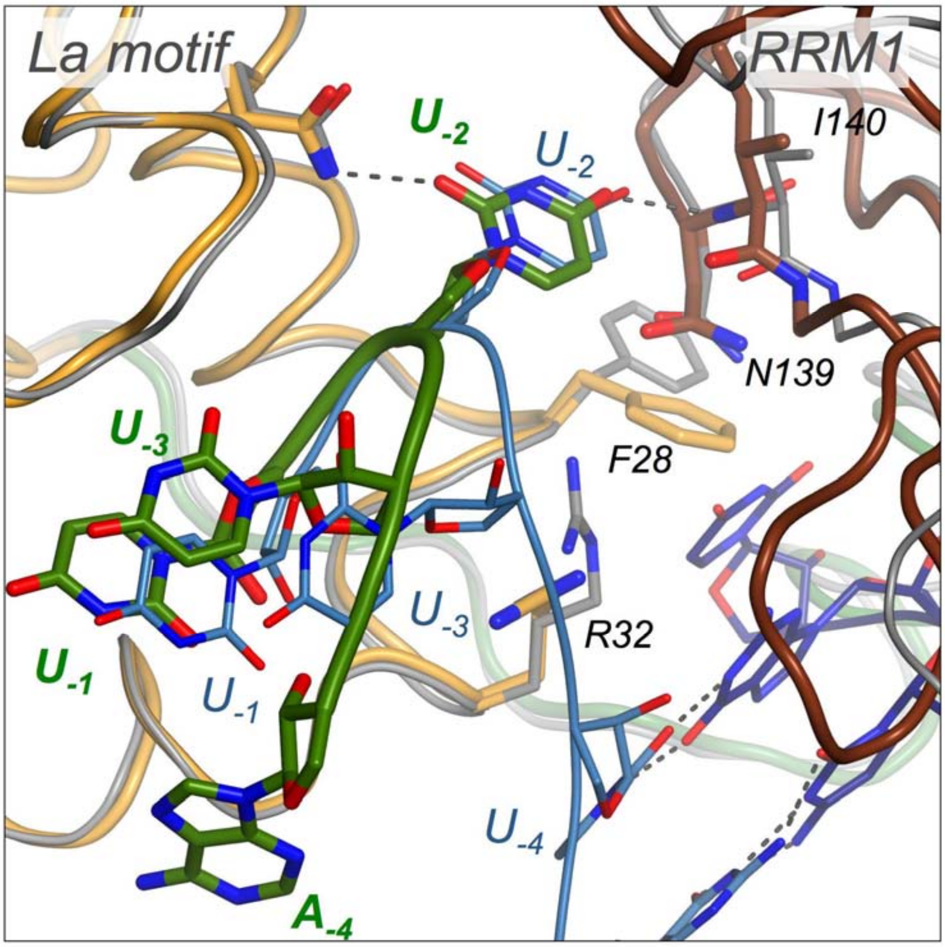


**Figure S1.** Comparison of the Inter-Domain Interface in the Presence of ssRNA and dsRNA Ligands

The structures were superposed using the coordinates of the La motif. The LaNTD:AUAAUUU complex is shown with the La motif coloured orange and RRM1 coloured brown; the carbon atoms of the AUAAUUU RNA are coloured green. The protein of the LaNTD:dsRNA complex is shown in grey; carbon atoms of the two strand of the dsRNA are coloured light and dark blue respectively. The positions of selected sidechains are indicated. Note the variation in the positions of F28 and R32 in the presence of different RNA ligands; in particular, the presence of a dsRNA ligand prevents F28 from stacking underneath N139.

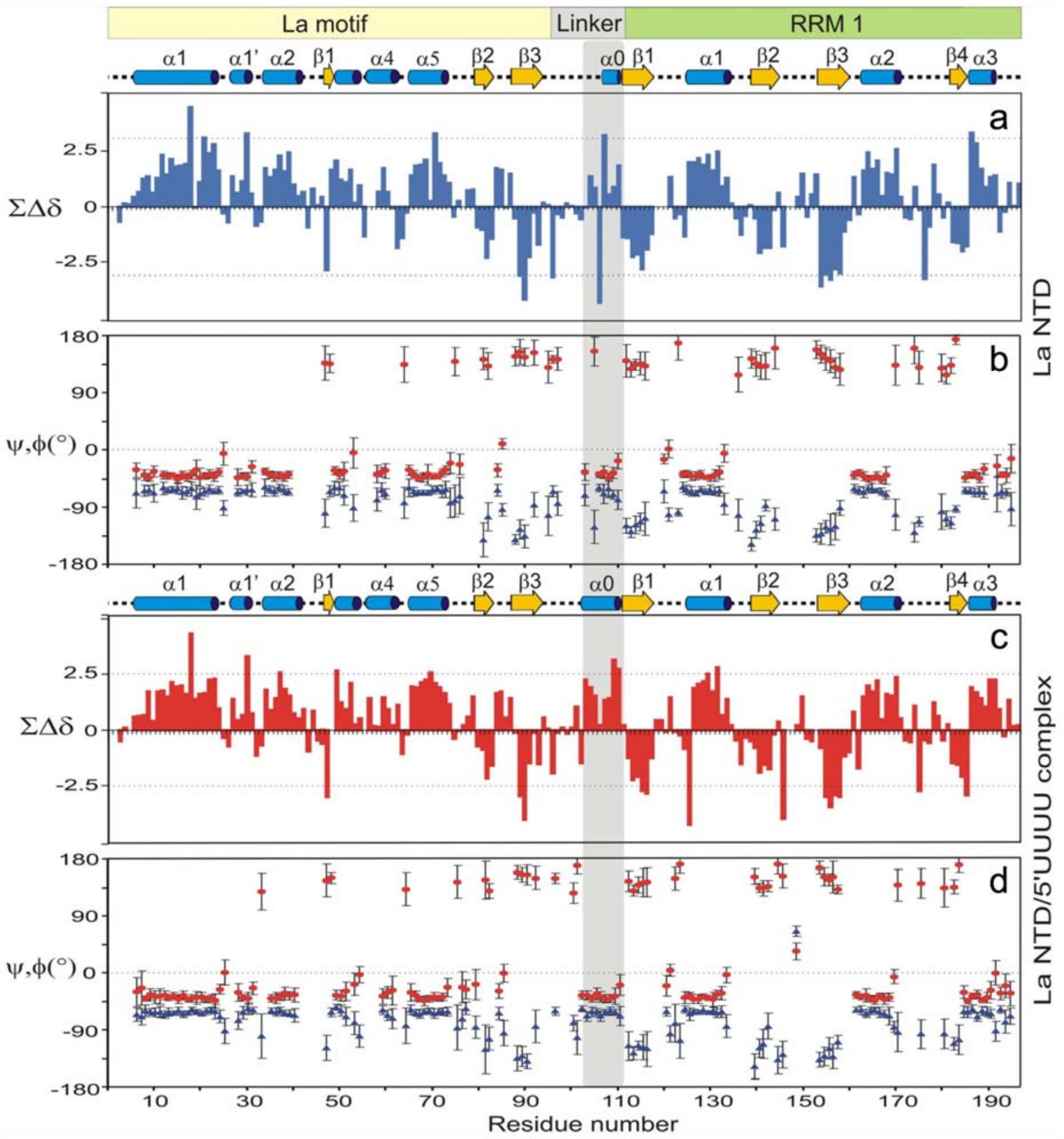


**Figure S2.** Secondary Structure Analysis of apo-LaNTD and in Complex with 5´-UUUU

(**a**) Chemical shift analysis (CSI) of apo-LaNTD reporting the chemical shift deviation from random coil obtained by subtracting the experimental values from the random coil chemical shifts and then deriving a weighted consensus value ($\Sigma\Delta\delta$) using ($4\Delta\delta_{H\alpha} + \Delta\delta_{C\beta} - \Delta\delta_{C\alpha} - \Delta\delta_{C'}$)/ number of assignments. Positive and negative values indicate α-helical or β-strand arrangement respectively. (**b**) Prediction of dihedral angles ϕ and ψ obtained by TALOS for apo La NTD. Only values with scores 9 or 10 are reported. (**c**) CSI for La NTD in complex

with 5'UUUU calculated as A. (**d**) Dihedral angles predicted by TALOS for La NTD/5'UUUU complex. Cartoon representation of the secondary structure delineation is reported for both apo and bound form. A shaded grey box highlights the variation in secondary structure experienced by residues 101-110 corresponding to the inter-domain linker. The error bars calculated by TALOS correspond to the standard deviation from the average angle for the center residue of the ten (or nine) best-fitting triplets in the database.

**Supplemental Experimental Procedures**

Protein samples for NMR were prepared in 20 mM Tris HCl pH 7.0, 100 mM KCl, 1 mM DTT (NMR buffer). LaNTD:RNA complexes were prepared at a protein:RNA molar ratio of 1:1.5. NMR spectra were recorded at 293 K on Varian Inova spectrometers operating at 14.1 and 18.8 T and Bruker Avance spectrometers operating at 14.1 and 16.4 T, processed using NMRPipe/NMRDraw (Delaglio et al., 1995) and analysed using XEASY (Bartels et al., 1995), NMRView (Johnson and Blevins, 1994) and Sparky (TD Goddard and DG Kneller, UCSF, USA). The full $^{1}$H, $^{15}$N and $^{13}$C resonance assignments for apo-LaNTD and complexed with UUUU and UCUU were obtained using standard protocols and will be reported elsewhere (Biomol. NMR Assignments, in press). Secondary structure elements were determined by the analysis of the chemical shifts of backbone atoms and $^{13}$C , by characteristic NOE patterns from the $^{15}$N-edited NOESY-HSQC and by backbone $\phi$ and $\psi$ dihedral angles obtained using TALOS software (Delaglio et al., 1995).

RNA titration experiments were performed by adding increasing amounts of unlabeled synthetic oligonucleotide to $^{15}$N-labeled LaNTD protein in NMR buffer. $^{1}$H-$^{15}$N HSQC spectra were recorded at RNA:protein mole ratios of 0.2:1, 0.5:1, 0.8:1, 1:1, 1.2:1, 1.5:1.

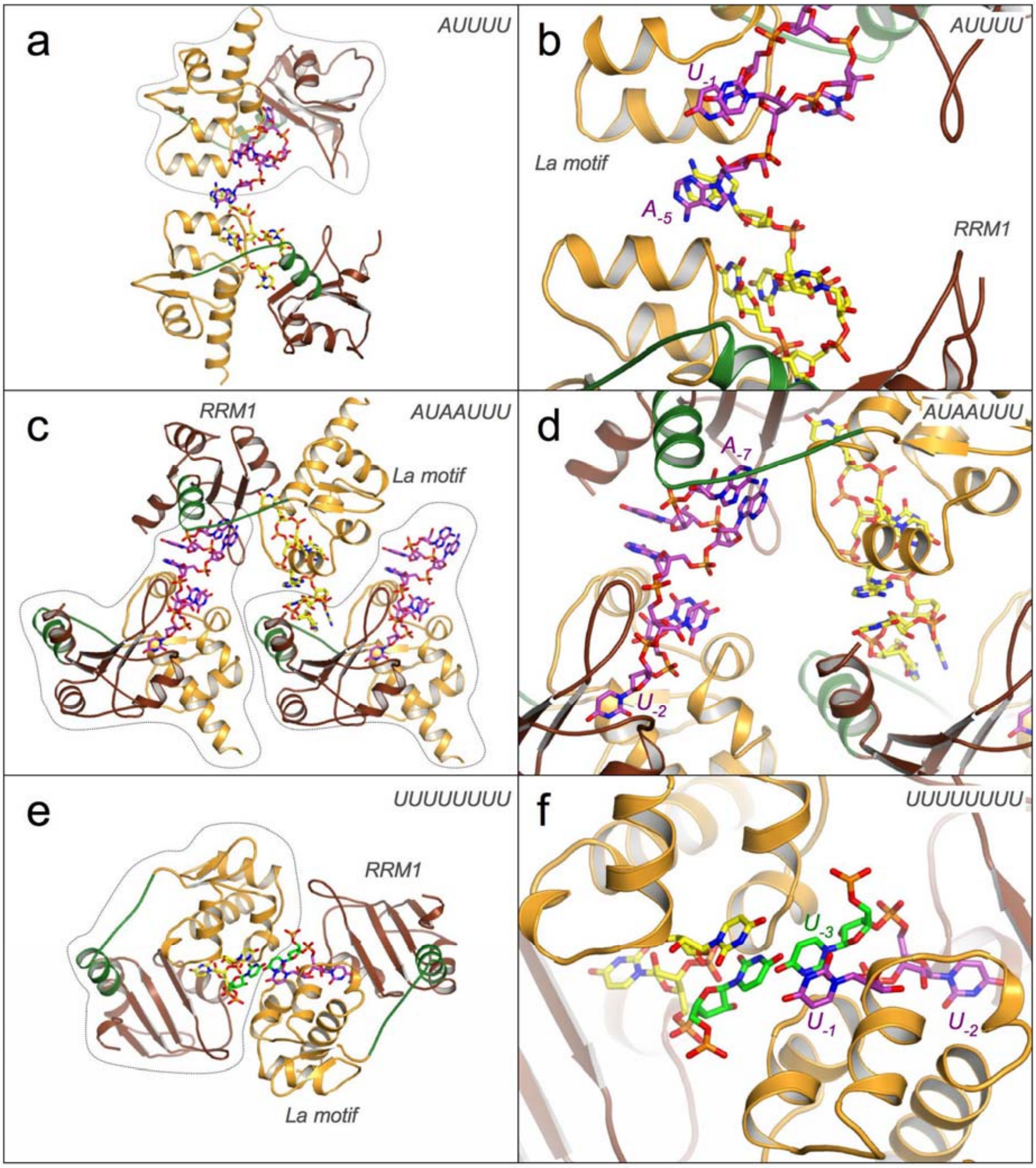


**Figure S3.** Crystal Packing Contacts Involving Bound RNA for Different LaNTD:RNA Complexes

**(a,b)** LaNTD:AUUUU complex. **(c,d)** LaNTD:AUAAUUU complex. **(e,f)** LaNTD:UUUUUUUU complex. The $U_{-3}$ nucleotide depicted with green carbon atoms was modelled into weak density but can only be partially occupied since there are steric clashed between this residue in symmetry-related molecules.

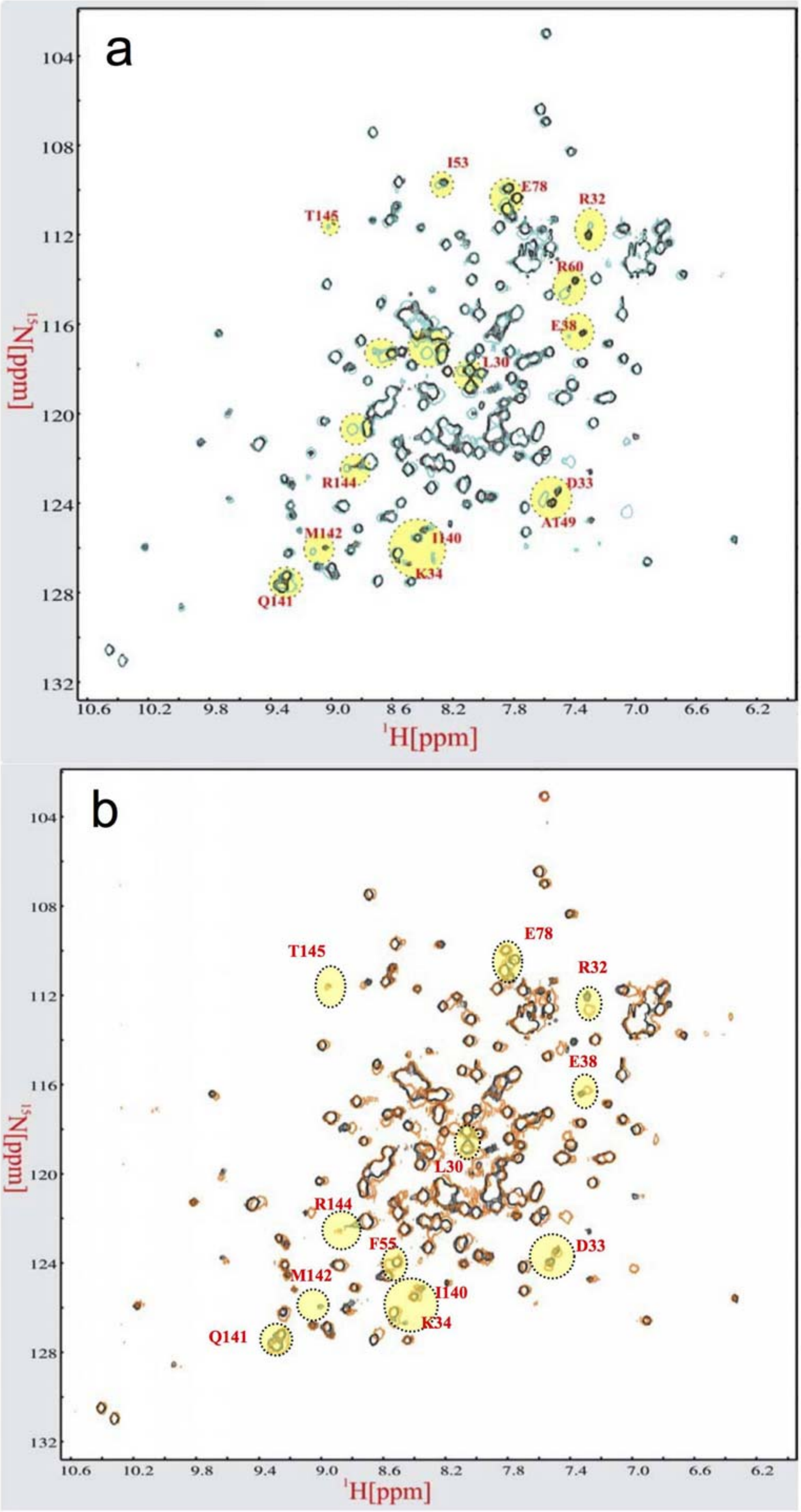

a
I53
E78
T145
R32
R60
E38
L30
R144
D33
A149
M142
I140
K34
Q141
15N[ppm]
1H[ppm]
b
T145
E78
R32
E38
L30
R144
F55
D33
M142
I140
K34
Q141
15N[ppm]
1H[ppm]

**Figure S4.** Comparative Chemical Shift Analysis of LaNTD Interactions with RNA

**(a)** [$^{1}$H-$^{15}$N] HSQC spectra from LaNTD:polyU10 (black) and LaNTD:AUUUU (cyan) are overlaid. **(b)** [$^{1}$H-$^{15}$N] HSQC spectra from LaNTD:polyU10 (black) and LaNTD:UCUU (orange) are overlaid. Regions with strong overlap could not be unambiguously assigned. Assigned residues exhibiting the most significant chemical shifts in each complex are labeled.

## Supplemental References


Bartels, C., Xia, T., Billeter, M., Güntert, P., and Wüthrich, K. (1995). The program XEASY for computer supported NMR spectral-analysis of biological macromolecules. J. Biomol. NMR *6*, 1-10.

Delaglio, F., Grzesiek, S., Vuister, G.W., Zhu, G., Pfeifer, J., and Bax, A. (1995). NMRPipe - a multidimensional spectral processing system based on Unix pipes. J. Biomol. NMR *6*, 277-293.

Johnson, B.A., and Blevins, R.A. (1994). NMRView - a computer-program for the visualization and analysis of NMR data. J. Biomol. NMR *4*, 603-614.

Sanfelice, D., Kelly, G., Curry, S., and Conte, M.R. (2008). NMR assignment of the N-terminal region of human La free and in complex with RNA. Biomol. NMR Assignments, in press.
